# International Nursing: Caring in Nursing Leadership—A Meta-ethnography From the Nurse Leader's Perspective

**DOI:** 10.1097/NAQ.0000000000000314

**Published:** 2018-09-05

**Authors:** Rita Solbakken, Elisabeth Bergdahl, Gudrun Rudolfsson, Terese Bondas

**Affiliations:** Faculty of Nursing and Health Sciences, Nord University, Bodø, Norway (Ms Solbakken and Drs Bergdahl, Rudolfsson, and Bondas); Department of Health Sciences, Division of Nursing, University West, Trollhättan, Sweden (Dr Rudolfsson).

**Keywords:** caring, caritative, leadership, meta-ethnography, nursing

## Abstract

To explore and derive new conceptual understanding of nurse leaders' experiences and perceptions of caring in nursing. Research question: What is caring in nursing leadership from the nurse leaders' perspectives? There is a paucity of theoretical studies of caring in nursing leadership. Noblit and Hares interpretative meta-ethnography was chosen because of its interpretative potential for theory development. *Caring in nursing leadership is a conscious movement between different “rooms” in the leader's “house” of leadership. This emerged as the metaphor that illustrates the core of caring in nursing leadership, presented in a tentative model*. There are 5 relation-based rooms: The “patient room,” where nurse leaders try to avoid patient suffering through their clinical presence; the “staff room,” where nurse leaders trust and respect each other and facilitate dialogue; the “superior's room,” where nurse leaders confirm peer relationships; the “secret room,” where the leaders' strength to hang on and persist is nurtured; and the “organizational room,” where limited resources are continuously being balanced. Caring in nursing leadership means nurturing and growing relationships to safeguard the best nursing care. This presupposes that leaders possess a consciousness of the different “rooms.” If rooms are not given equal attention, movement stops, symbolizing that caring in leadership stops as well. One room cannot be given so much attention that others are neglected. Leaders need solid competence in nursing leadership to balance multiple demands in organizations; otherwise, their perceptiveness and the priority of “ministering to the patients” can be blurred.

NURSE LEADERS play a key role in articulating the uniqueness of nursing in complex health care systems.^1^ They are responsible for safeguarding the best care and caring for the patients.^2^,^3^ Nurses have a long tradition of leading health services. They are known by colleagues and the public as leaders of a caring profession.

This meta-ethnography reports a new conceptualization of what caring in nursing leadership is from an insider's perspective. It is based on experiences from nurse leaders themselves. The articles included here use different concepts to describe participants. However, the majority of the articles present the first-line nurse managers' perspective. These are the leaders working closely with patients and personnel, often with a tripartite responsibility for personnel, finances, and patient care. In this article, we use the term “nurse leader” for these individuals.

Demands on nurse leaders are multiple and sometimes contradictory. Nurse leaders are expected to implement organizational and political reforms, and the importance of their strong leadership is emphasized in government documents. While some of these mandates affect health care positively, others have negative impacts. Nurse leaders must work to mitigate those that pose a risk to humanity and that threaten to diminish the focus on patients as unique human beings. The most important goal of nurse leaders is to safeguard the welfare of suffering patients. To do this requires a relationship between the nurse leader and the nursing staff to create conditions where nurses can care for their patients and families.^1^,^3^–^5^

Health care organizations are constantly changing. Demographic changes, including an ever-increasing number of elderly suffering from chronic diseases, require constant restructurings. Business-orientated leadership models also influence health care. For example, in Norway, the New Public Management (NPM) objectives are mainly aimed at reducing costs through budget cuts, restructuring, and downsizing of the staff.^2^,^3^,^6^ NPM focuses on financial measures but neglects the qualitative areas of care that might not be easy to measure and fit into the NPM concept. As some authors have noted, change in traditional health care roles is necessary if service delivery is to be improved.^7^ In response, other authors note that there is a current shift from business-orientated and hierarchical leadership models toward models emphasizing leadership as relationships to others.^8^ This change holds the potential to bring a new lens for viewing leadership and caring.

Transformational,^9^ servant,^10^ and authe-ntic^11^ leadership theories have become popular. The main distinctions are that transformational theories tend to focus on organizational objectives, servant theories focus on the staff, and authentic theories focus on the leaders themselves.^12^,^13^ A newer theory, caritative leadership, contrasts these theories by its emphasis on the patient and his or her care needs.^2^ Caring is increasingly recognized and described as the moral ideal and essence of nursing.^14^–^16^ That is, it is a moral commitment toward protection, enhancement, and preservation of human dignity. Caring may be understood as the human expression of, respect for, and response to wholeness. This is defined as an active engagement in the person-to-person process of being and becoming.^17^ Mayeroff^18^ expresses the meaning of being a caring person by referring to the caring relationship, as experienced through caring actions that include being with, and “being for,” a person who needs care.

Caring in nursing leadership can be perceived when nursing leaders facilitate good care on the nursing units. However, nursing cannot be defined by caring alone.^19^,^20^ Nurse leaders and staff nurses need to combine care with knowledge.^2^ Otherwise, the importance of nurses' knowledge and nursing as a scientific discipline will be undermined.^21^ Patients suffer when either knowledge or caring is missing. Instead of receiving the care they should be able to expect, they sometimes encounter caregivers who violate their dignity, are nonchalant, and even neglectful.^22^,^23^ To decrease suffering, nurse leaders have an inherent duty to provide environments that encourage and nurture care for patients.^2^,^24^

Leading complex organizations with accountability for high-quality nursing despite diminished resources can lead to hopelessness and a lack of commitment to the traditional ideals of nursing as a growing profession.^2^,^17^,^25^ To combine caring with fiscal stewardship is challenging and is perceived as time-consuming for nurse leaders. However, both are essential duties that nurse leaders must balance.^6^,^24^ By learning the core behaviors of caring while working in environments that support them in balancing their accountabilities, nurse leaders are able to make better ethical decisions that support care for the patient, the individual staff member, and the organization.^2^,^26^–^28^ These caring behaviors include mutual respect, fairness,^29^ and commitment.^2^,^15^,^30^,^31^

The interest in caring and caring sciences in nursing has increased because of the advances in nursing science. While it is easy to understand its importance to the role of the bedside nurse, the essence of caring in leadership is difficult to conceptualize. However, caring needs to be recognized as an important competency for nurse leaders. One theory of leadership is particularly pertinent to nurse leaders. Known as caritative leadership, it is based on the motive of caritas and is derived from the concept of humanistic caring and service to humanity.^2^,^32^ Its main tenet is that ministering to the patient^2^ contributes to an existential awareness about personal and professional meaning and purpose. This creates a more healing work environment.^25^,^26^,^32^

There are a number of studies on caring in nursing leadership reported in the literature. They are presented from several perspectives.^28^–^30^ This article is based on a study undertaken to examine caring from the perspectives of nurse leaders themselves.

The aim of this meta-ethnography study was to explore and derive a new conceptual understanding of nurse leaders' experiences of caring in nursing leadership. The research question was as follows: What is caring in nursing leadership from the nurse leaders' perspective?

## METHODS

There are several current methods for metasynthesis, and they continue to evolve.^33^ Metasynthesis is done by searching for studies to answer a research question, followed by evaluating the studies, breaking up the different parts of the reported data in the studies, and examining the whole of each study and data to undergo analysis, synthesis, and interpretation.^34^

“Meta-ethnography” is a generic term for review approaches that employ an interpretive form of synthesis based on qualitative studies of a phenomenon of interest.^35^,^36^ It aims to broaden our knowledge by reusing existing qualitative studies by transforming them into analytic and theoretical concepts.^35^ This moves beyond the original study reports to situate their findings, historically, define them for the present, and chart future directions in that domain.^37^ This approach was chosen as a methodology to possibly create a new understanding of the phenomenon of caring in nursing leadership.

This approach is characterized by interpretation and creation of new knowledge, not aggregation of findings.^35^,^36^ Therefore, it is more than the sum of the parts in that a novel interpretation of findings is offered. Meta-ethnography offers a greater understanding when the goal is combining and comparing findings that are more substantive in depth and breadth than those from individual studies.^34^ The rationale for such studies is that they potentially create a knowledge base for qualitative studies that enable evidence-based practice while developing theoretical models to guide evidence-based practice and decision-making.^33^,^38^

### Criteria and search strategy

The inclusion criteria for this meta-ethnography were peer-reviewed, empirical studies of all qualitative methodologies that focused on caring in leadership/management in any health care context and that were published in scientific journals found in online English or Scandinavian databases. There were no temporal or geographical limitations. Only studies with nurse leaders as informants, or studies with mixed informants or mixed methods where qualitative findings from the leader's perspective could be separated, were included.

The exclusion criteria were quantitative studies; findings from the staff nurse's or patient's point of view; reports of studies where perceptions from nurse leaders could not be separated from other findings; theoretical and review articles; and master's theses and dissertations.

**Figure 1.**
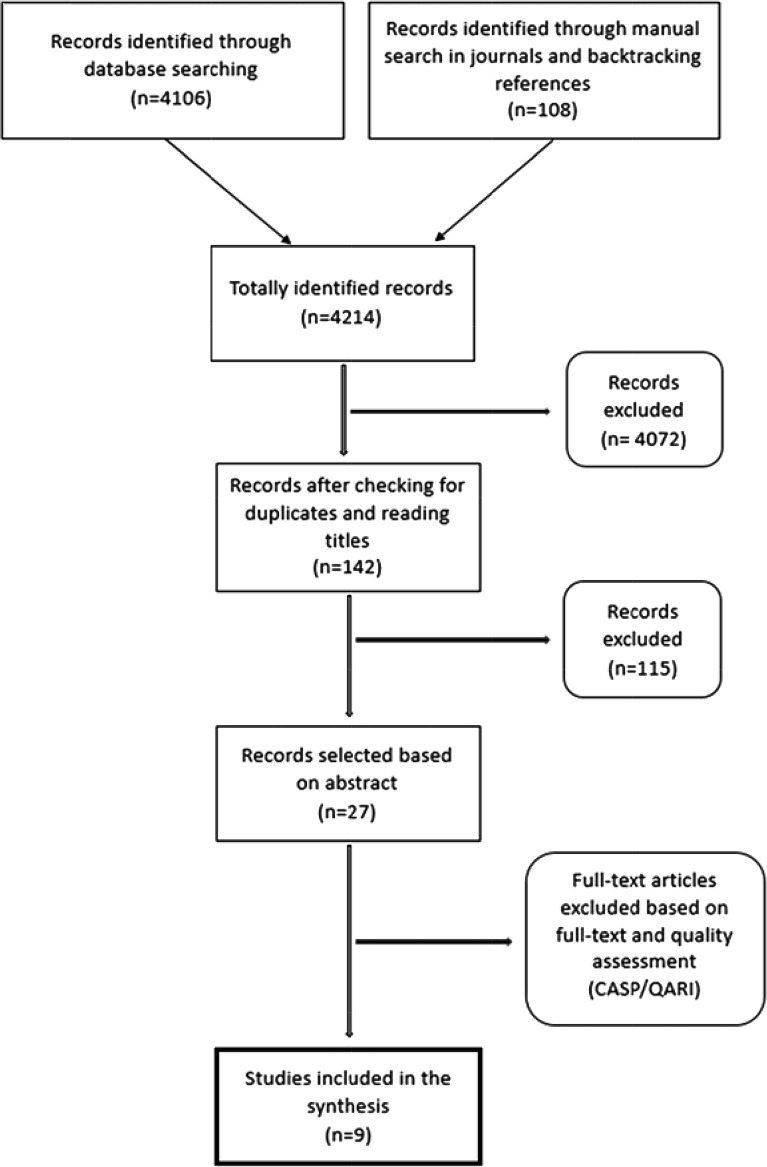
Search strategy and results of different phases.

Relevant databases and key terms in English were found with help from experienced librarians. Main key search terms were “nursing leader,” “care,” “caring leadership,” “caring culture,” “caritative leadership,” and “qualitative” in various combinations. The following databases were searched: CINAHL, Wiley, Science Direct, Google Scholar, Scopus, and PubMed. To ensure coverage, references were backtracked and manual searches were completed in 4 relevant journals: *Journal of Nursing Management, Journal of Nursing Administration, International Journal for Human Caring*, and *Nursing Administration Quarterly*. Previous literature reviews were searched, and author and ancestry searches were performed to access studies not identified through the database search. Databases and journals were shared among the authors. Each author conducted all phases of the search process independently. To ensure agreement on the search process, inclusion of studies, and appraisal of quality, all authors attended regular meetings via Skype. When uncertainty at any point in the process occurred, it was discussed within the research group until an agreement was reached. The search strategy, databases, journals, results of each step, and the selection process are presented in Table 1 and Figure 1. Characteristics of included studies are summarized in Table 2.

**Table 1.** Search Strategy and Results of Different Phases

Years	Database	Total	Selections Based On Title	Selections Based On Abstract	Selections Based On Full Text and Inclusion Criteria (CASP/QARI)
All	CINAHL	2993	14	2	0
	Wiley	11	6	5	4
	Science Direct	436	18	3	0
	Google Scholar	65	25	2	2
	Scopus	593	39	12	2
	PubMed	8	2	1	0
	Manual search in journals by backtracking of references	108	38	2	1
	Total	4214	142	27	9

**Table 2.** Articles Included in the Meta-ethnography

Author(s) (Year Published), Country		Participants, Gender, Age	Time Practicing as Leader	Context	Aim	Qualitative Research Design	Data Collection Data Analysis
1.	Bondas (2009), **Finland**	**65 nurse managers** Both (60 F/5 M) **25-55 y**	**3 mo-26 y**	Rural/urban areas Small health care centers and hospitals	To gain an understanding of the first-line nurse managers in their experiences in the development of nursing care as part of a wider research program	Grounded theory	Narratives written at the beginning of 5 different leadership courses, based on open-ended questions narratives
2.	Orvik et al (2015), **Norway**	**10 ward managers** Both (9 F/1 M) **Not specified**	**1-12 y**	6 Hospitals in 3 different health regions	To explore and describe the value squeezes experienced by ward managers in connection with quality management in hospital wards	Descriptive qualitative	Semistructured interviews
3.	Rosengren and Bondas (2010), **Sweden**	**2 nurse leaders** Female **36-37 y**	**18-20 y**	1 Intensive care unit	To describe 2 nurse managers' experience of working together as equal partners within a shared leadership model	Grounded theory	12 interviews; 6 interviews with each manager
4.	Rudolfsson and Flessner (2012), **Sweden**	**10 nurse leaders** Both (8 F/2 M) **41-58 y**	**1-14 y**	10 operating departments, various hospitals	To capture and interpret meanings of suffering from the perspective of perioperative nurse leaders	Hermeneutic	Individual interviews with open-ended questions
5.	Rudolfsson et al (2007), **Sweden**	**10 nurse leaders** Both (8 F/2 M) **41-58 y**	**1-14 y**	10 operating departments, various hospitals	To achieve a more complete and differentiated understanding of what constitutes caring in the perioperative culture as well as their knowledge and responsibility for the development of caring	Philosophical hermeneutics	Interviews
6.	Salmela et al (2012), **Finland**	17 nurse leaders; 14 head nurses, and 3 directors of nursing Female 42-61 y	**Not specified** *Work experience: 2-26 y*	1 primary health care center and 1 local hospital	To explore how nurse leaders describes and understand their main task and role during a change process	Phenomenological-hermeneutic	In-depth interviews
7.	Salmela et al (2011), **Finland**	17 nurse leaders; 14 head nurses, and 3 directors of nursing Female 42-69 y hospital/42-61 y health care center	**2-31 y hospital/3-31 y health care center**	1 local hospital and 1 primary health care center	To achieve a more profound understanding of nurse leaders' perceptions of an approaching organizational change	Three-dimensional hermeneutic model of interpretation and understanding	Semistructured in-depth interviews
8.	Sørensen et al (2011), “**Denmark**	12 leading nurses; 5 first-line nurses (in sections) and 7 nurses at the department level Not specified 39-57 y	**Not specified** *At least 3-4 y*	6 hospitals in 2 counties	Exploring the negotiation between nursing and leadership in hospital head nurses' leadership practice.	Phenomenological, hermeneutical	Participant observation, semistructured interviews, field notes, and ethnographic interview techniques
9.	Uhrenfeldt and Hall (2009), **Denmark**	10 nurse leaders Both 40-63 y	**3-19 y**	2 hospitals	To investigate proficient first-line leaders' caring for the nursing staff	Hermeneutic, phenomenology	Individual, semistructured interviews

A lack of consensus concerning quality in qualitative research and the use of checklists in metasynthesis is controversial.^34^,^39^–^41^ Some experts believe that studies should not be excluded for quality reasons, because there is a wide variation in conceptions of the criteria for good quality, and there is a risk of excluding research results from missing studies.^42^ Others argue strongly for appraising studies.^43^ It was agreed that studies should be quality checked, and the authors chose to combine research quality assessment tools CASP^44^ and QARI.^45^ The full-text articles considered for inclusion were shared among the authors. One author did the critical appraisal of each article and then presented and made the case for inclusion in the study to the other authors. When there was disagreement about inclusion, another author did a second appraisal to determine whether the article should be included or excluded. Four articles failed this assessment process (see Table 3). Nine articles were eventually chosen for inclusion in this meta-ethnography.

**Table 3.** Excluded Studies: Details of Quality Assessment According to CASP/QARI

		“Nurse Leaders' Responsibilities in Supporting Nurses Experiencing Difficult Situations in Clinical Nursing” (Hankavou and Lindstrom, 2014)^46^		“Authentic and Congruent Leadership Providing Excellent Work Environment in Palliative Care” (Johansson et al, 2011)^47^		“Demystifying Ward Nurse Managers Approach to Managing Change” (Moen and Core, 2012)^48^		“Walking a Tight Rope: An Investigation of Nurse Managers Work Stressor and Coping Experiences” (Udo and Dean, 2012)^49^	
Quality Appraisal CASP	Quality Appraisal QARI	CASP/QARI		CASP/QARI		CASP/QARI		CASP/QARI	
Clear statement of aim?	Is there congruity between the stated philosophical perspective and the research methodology?	No	Unclear	Yes	N/A	Yes	Yes	Yes	Yes
Is qualitative methodology appropriate?	Is there congruity between the research methodology and the research question or objectives?	Cannot tell	No	Yes	No	Yes	Yes	Yes	Yes
Was the research design appropriate to address the aims of the research?	Is there congruity between the research methodology and the methods used to collect data?	No	Yes	No	Yes	Yes	Yes	Yes	Yes
Was the recruitment strategy appropriate to the aims of the research?	Is there congruity between the research methodology and the presentation and analysis of data?	Cannot tell	No	No	Unclear	Yes	Yes	Yes	Yes
Were data collected in a way that addressed the research issue?	Is there congruity between the research methodology and the interpretation of results?	Yes	Yes	No	Unclear	Yes	Yes	Yes	Yes
Has the relationship between the researcher and participants been adequately considered?	Is there a statement locating the researcher culturally or theoretically?	No	No	No	No	No	No	Cannot tell	No
Have ethical issues been taken into consideration?	Is the influence of the researcher on the research, and vice versa, addressed?	Yes	No	Yes	No	Yes	No	Yes	No
Were the data sufficiently rigorous?	Are participants, and their voices, adequately presented?	No	No	No	Yes	Yes	Yes	Cannot tell	Yes
Is there a clear statement of findings?	Is the research according to current criteria or, for recent studies, and is there evidence of ethical approval by an appropriate body?	No	Yes	No	Yes		Yes	Yes	Yes
How valuable is the research?	Do the conclusions drawn in the research report flow from the analysis, or interpretation, of the data?	Not	No	Not	No	Not	Yes	Yes	Yes

### Data abstraction and synthesis process

The interpretative process that followed the selection phase was based on the approach described by Noblit and Hare.^35^ Each author independently read through the 9 articles several times to get an understanding of the whole study and of the specific study details. In the next phase, the studies were analyzed by juxtaposing the findings to make a decision on how the studies were related. The authors found them to be largely analogous and compatible. Studies were categorized by their key terms, concepts, and findings and then translated into each other. This means that the findings of each study were compared and contrasted, a process Noblit and Hare^35^ call “translation,” as a key phase in the meta-ethnography. Preservation of the meaning from the original articles was important. To identify homogeneity or incongruity between the themes, the articles were juxtaposed systematically. The subthemes and then the main themes were synthesized.^35^

A second level of synthesis, the metaphoric “rooms,” emerged in the analysis process, developed, and validated by returning to the previous phases. The metaphor was then developed. As a result, a new tentative model of caring in leadership was created. The first author did the analysis and synthesis. All 4 authors met and discussed their understanding of the themes and metaphors regularly to validate the process and the findings.

Because of differing education and varied professional and leader experiences in Nordic countries, as well as previous research on caring (particularly on caring in nursing and nursing leadership), the research team took a critical approach to preunderstandings.

## FINDINGS

Fifteen subthemes concerning leadership caring emerged from the data. These were synthesized into 5 main themes: alleviating suffering by clinical presence; trusting and respecting, and facilitating dialogue; needing confirming relations; having the strength to hang on and persist; and balancing limited resources (Table 4). The main themes were abstracted into metaphoric mental and physical rooms as part of the leader's house of caring: the patient room, the staff room, the superior's room, the leader's secret room, and the organizational room. A metaphor was developed: *Caring in nursing leadership is a conscious movement between different rooms ministering to the patients*. This illustrated the core of caring in nursing leadership, which was visualized as a tentative model. Caring in leadership reflects upon 2 themes: consciousness and movement. Both are necessary to keep caring alive. In this model (model 1), organizations consist of different metaphoric rooms, each representing a separate need to be taken into account in caring leadership. It becomes pivotal that caring in leadership is about leading relationships. It presupposes that leaders are conscious of movement between these different rooms, where movement is a metaphor and involves an effort from leaders to undertake caring actions. If the rooms do not get equal attention or if there is an imbalance between the rooms, the movement stops. This symbolizes that caring in leadership stops as well. The imbalance can be due to the leader's own ability or to challenging external conditions. Leaders may also feel “safe” in one of the rooms when caring prevails. On the contrary, they may be frightened to enter another room. The findings of this meta-ethnography are presented according to the model and the metaphors. They are not mutually exclusive and should therefore be read as related to each other.

**Table 4.** Subthemes and Themes

Subthemes	Articles	Themes
Developing an open, esthetic, and caring culture	50–53	*Trusting and respecting, facilitating dialogue*
Teaching, inspiring, guiding, and empowering nurses to develop their nursing care competence in collaboration	50,51,53,54	
Facilitating trust and respect to avoid conflicts and suffering to ensure good working conditions	50,51,53–56	
Creating arenas for open and mutual dialogue to guide the best nursing care	50,51,53–57	
Challenges for creating dialogues	55,57	
Clinical presence to know about and protect the patients	53,54,58	*Avoiding suffering by clinical presence*
Protecting the patient from suffering by empowering nurses to improve care	50,53,55,57,58	
Teaching and guiding the nurses to develop care in collaboration	53,54	
Motivating and expecting the nurses to always have the patients' benefit in sight	50,51,56–58	
Trying to create collaboration between professions and units to benefit patient care	57,58	
Focusing on patients' well-being and developing the best possible care in their units	50–55	
Accepting and balancing limited resources by prioritizing	50,54,56	*Balancing limited resources*
Leading by being a good role model	50–54,55,57	
Leading demands and personal strength to be passionate toward the mission	50,52,55–58	*Having the strength to hang on and persist*
Relation and competence neglect from the superiors causes insecurity concerning tasks and roles; this gives a feeling of being excluded	50,52,55–58	*Needing confirming relations*

Dialoging, inspiring, teaching, motivating, and guiding emerge as skills that are required in caring leadership. These are tools needed to facilitate collaboration at all levels in the organization and are closely linked to cooperation among team members. Daily conferences with the staff are emphasized by caring leaders to discuss nursing care matters, situations, and workloads. A pivotal task of the leader is to create arenas for dialogue, thereby facilitating a unit where open discussions occur.^50^–^52^,^54^ This gives the staff and leaders opportunities to inform each other. Staff members are able to express opinions while being listened to. Problem solving is done through ongoing, open, and honest communication. By dedicating this time to dialogue, leaders show their care for the staff and indirectly support patient care.^50^,^51^,^53^–^55^,^57^

A collaborative relationship with the staff is perceived as ideal. Nevertheless, nurse leaders sometimes need to limit discussions to protect themselves from harsh comments.^50^ In addition, nurse leaders teach their staff to listen and learn from each other^53^ in order to broaden their perspectives of patient care.^54^ Participating on units as part of a team is essential for gaining a greater understanding of patients, staff, and the organization, which supports the provision of high-quality care.^50^–^58^ This quality can also be ensured by nurse leaders who move between the rooms where different perspectives on care are experienced and understood.

### The patient room

A core characteristic in the study findings is that every task a nurse leader accomplishes is intended to support the best possible patient care.^50^–^58^ Organization changes should emanate from patients' needs, and nurse leaders must represent and speak for patients in change discussions.^51^,^53^ Nurse leaders communicate a desire for the best performance of staff members but sometimes struggle to keep sight of the patient in their leadership. They see themselves as facilitators and role models for raising the quality of care.^50^,^53^,^54^,^57^ Therefore, nurse leaders strive to prioritize their duty to know patients, protect them from harm, and influence the development of procedures supporting the best possible care.^50^–^55^,^58^ Nurse leaders are concerned about shortcomings in patient care and the underreporting of deviation from quality.^50^,^57^ Dedication to superior nursing care seems deeply rooted in the nurse leader and is a driving force for him or her in becoming a leader.^54^,^58^ Nevertheless, one of the studies indicated that some leaders have different perspectives on the importance of a clinical presence for the nurse leader.^58^ Some nurse leaders assist in bedside tasks to resource patient care demands^54^,^58^; others are complementary experts in nursing.^54^ Clinical presence is often prioritized above administration duties because of lack of time. This may result in a fear that this affects their perception of what the patients need.

### The staff room

Findings indicate being a nurse leader means relationship building. Nurse leaders describe a feeling of responsibility for creating good working conditions for their staff so that staff members are able to give the patients the best care possible.^50^–^53^ “Openness” of the leader is emphasized as a key to develop good working conditions and is regarded a managerial and human responsibility.^53^,^57^ Findings suggest that nurse leaders want to create cultures characterized by openness, trust, safety, and flexibility. Frequent discussions should occur, and staff members should be given the opportunity to express their views and to be listened to^50^,^51^ in order to retain sight of the patient.^53^,^56^

The study reflects nurse leaders' feelings of responsibility for their staff's well-being. They care for their staff by involving them in problem solving and in the decision-making processes. This includes being a coach, supporting and appreciating staff achievements, as well as standing up for their units.^50^,^53^,^54^ Nurse leaders feel responsible for handling unforeseen tasks so that the credit reflects well on their staff.^54^,^56^

Dealing with conflict is a leadership issue that cannot be delegated. Nurse leaders expect that they, as well as their staff, must contribute to respectful and trustful relationships, mitigate conflicts, and minimize suffering. They see valuing personnel as equal to valuing nursing care.^50^,^51^ Seeing and treating each nurse as an individual are anticipated to create a safe and caring atmosphere on the unit.^50^,^51^,^53^–^55^,^58^ This presupposes that nurse leaders are able to be team players, who demonstrate transparency when sharing information, giving instructions, or prioritizing work.^51^,^55^ Nurses must be willing to allow the nurse leader into “their” room so that the nurse leader becomes a part of the team in a shared room.

As different methods in health care develop, nurse leaders expect nurses to play an active part in the development of care.^53^,^54^ Nurse leaders use a wide range of approaches, such as teaching, inspiring, guiding, and empowering, to encourage the nurses to develop their nursing care competence.^50^,^51^,^53^,^54^ Staff competence in nursing care is emphasized as pivotal to quality.^50^,^51^,^53^,^54^ By knowing each staff member and his or her competencies, nurse leaders are able to pursue individual plans to increase knowledge and training of the entire team.^50^,^54^ Caring leaders give staff members the time, space, support, and encouragement to enhance their individual skills.^50^,^51^,^53^ This requires continuous planning for staff education; the giving and receiving of feedback on quality of nursing; and support and inspiration for nurses to develop caring by spreading knowledge that benefits patients.^50^,^51^ Findings suggest that both theoretical knowledge and caring skills are required.^50^,^51^ A discrepancy in perception among nurse leaders on the value of theory from nursing science was found to be prevalent.^50^,^56^ A variety of thoughts on both competence and caring are evident in the literature. Leaders are frustrated and find it difficult to deal with the staff who lack empathy for others, including patients. They want quality control systems based on ethical norms and not just legal or regulatory requirements. They know that leadership requires maintaining a balance between controlling and trusting staff members.^50^–^52^,^57^ They are also concerned about underreporting of deviation from care standards and staff members who do not report errors.^57^ Nurse leaders associate competence with reporting deviation, and it is clear that they perceive high competence to be a key factor in quality patient care.^50^–^55^,^57^,^58^

### The superior's room

Nurse leaders struggle to keep up with their workload while fulfilling the assumed expectations of both their staff and superiors. Acknowledgment from and confirming relations with their superiors seem essential for nurse leaders' self-confidence and well-being. They describe the need to be valued by others. However, they report that they often lack support from both their staff and their managers, which results in a feeling of devaluation and loneliness. From the point of view of nurse leaders, neither staff nor superiors understand their jobs and the tasks of management are not given the status they deserve.^56^,^58^ The term “unfair” is used when efficiency and high-quality work are not appreciated and when their decisions or competence are questioned.^50^,^56^,^58^

### The leader's secret and lonely room

Being a nurse leader is presumed to be a demanding position, combining a heavy workload and high personal stress. Findings indicate that choosing to be a nurse leader should be a deliberate and carefully considered choice.^50^,^55^,^57^ Nurse leaders need to be integrated into teams at 3 levels: on their own units; with peers' who manage other units; and with leaders across the entire organization. This can lead to conflicts of loyalties due to a need to balance differing requirements from these groups.^57^ Nurse leaders need to make nursing visible. They must conceive ideas for the further development of nursing care and then have the courage to fight for their ideas.^50^,^58^ They see leadership as more than a job. Their role is to participate in important work for the sake of human beings, and they express the will to do this despite challenges associated with the task and the expectation that they balance incompatible requirements.^55^,^57^ While taking this role on, they know that combining family and leadership is difficult.^55^

Nurse leaders emphasize the need for a combination of personal, clinical, and administrative skills in leadership.^50^,^55^ A long list of personal skills has been identified and is assumed to be a prerequisite for leadership. These include a positive attitude, an ability to collaborate, a persistent respect for others' strengths and weaknesses, flexibility in handling the unforeseen, a vision for developing quality care models, and assertiveness combined with patience.^51^,^53^,^57^

Nurse leaders are challenged to prioritize use of their time. Leadership in nursing has been compared with constant firefighting, rather than a result of strategic planning and professional development of care. Nurse leaders feel a need for sharing and balancing their time between safeguarding the patient and tending to the nurses' welfare.^53^,^57^,^58^

Nurse leaders experience regular threats from other professions pushing to take over leadership of nursing care, which results in the nursing perspective being overlooked. This makes collaboration between professions and units challenging. Despite this, nurse leaders continue in their endeavors to create cross-professional, respectful arenas for collaboration in the interest of benefitting better patient care.^50^,^56^ While politically motivated economic cutbacks can be burdensome, nurse leaders find ways to protect patient care quality. Some have merged units and created new units with a common culture of caring to offer patients the best possible care with constrained resources.^50^,^55^

Nurse leaders are considered middle management placed between the staff and management. There is some uncertainty to what extent nurse leaders prefer to align themselves with the staff or with management. Those who are accepted by the staff as part of the team can experience feelings of distress.^50^,^55^,^58^ Some characterize themselves as well integrated with the staff. These managers get feedback that staff members are not experiencing clear leadership and have differently accepted the manager's leadership. Nurse leaders identifying themselves with their staff often prioritize clinical work but sometimes report that staff members question the nurse leader's knowledge and clinical skills, while lacking an understanding of the nature of managerial work. Nurse leaders work alone to a large degree and must make many decisions without support from others. This relative isolation can give nurse leaders a feeling of being excluded and not being “one of the gang.”^50^,^55^–^58^

### The organizational room

The structural framework that encloses the interior rooms where leadership takes place is the organizational room. Balancing and coordinating limited resources are a part of a leader's role. Nurse leaders prioritize, make strategic choices, and lead activities so that the staff can work as effectively as possible. Multiple leadership tasks force nurse leaders to strictly prioritize their own time for maximum efficiency.^50^,^54^,^58^ Their professional standards are squeezed between ethical standards, economical resources, and quality of care.^50^,^53^,^58^ The connection between shortages of resources and diminished quality of care is experienced as a difficult issue for nurse leaders. If their professional standards cannot be met because of lack of resources, they are distressed and ashamed. Nurse leaders ask nurses to increase efficiency in their struggle to maintain these patient care standards even when some nurse leaders choose to adjust their standards instead of reporting deviations to upper management.^50^,^56^ Nurse leaders are also striving to obey regulations despite limited resources. They are eager to do their best for patients and staff members under constrained conditions but feel responsible for the consequences when they are forced to act in a way that can be unfavorable for patients or the staff.^50^,^55^,^58^

## DISCUSSION

The authors developed metaphors based on the synthesis of these main themes: balancing limited resources; avoiding suffering by a clinical presence; trusting, respecting, and facilitating dialogue; needing confirming relations; and having the strength to hang on and persist. These themes were interpreted and assigned (metaphorically) as rooms for the patients, staff, superiors, leaders, and organizations. Rooms were visualized to show their connections. This is a tentative, theoretical model for understanding of caring in nursing leadership. The continuous movement between different rooms revolves around the nursing duty to patient care. It symbolizes and illustrates caring in leadership from leaders' personal perspective.

Caring leadership involves conscious movement between different rooms. A caring leader should make an effort to balance time spent in each room, with the patients' best interests in mind. These metaphoric rooms coincide with managerial roles for nurse leaders, with the exception of the leader's secret room at the center of the model. In this room, the nurse leader reflects and decides between entries to other rooms. Nurse leaders may prefer certain rooms. Every room can grow or diminish depending on whether it is prioritized or neglected. An imbalance between room activities indicates that movement between rooms has stalled. This creates an environment where patient care may be compromised. Conceptualizing caring in leadership as appropriate, balanced movement between these imagined rooms is a new model for balanced leadership.

This model is a framework for caring leadership. Nurse leaders are not only administrators but also individuals who care. Leaders strive to provide the best care possible. This is related to the ideal of caritative leadership^2^ when alleviation of patients' suffering is the main focus. Caritative leadership is typified by human love and mercy, and its main tenet is ministering to patients. Its 5 themes are as follows: the caritas motive; dignity; measurement of health; meaning of health care; and participation in the caring culture. Caritative leadership consists of developing, guiding, planning, organizing, reporting, directing, staffing, budgeting, coordinating, decision-making, and evaluating,^2^ which coincide with the findings in this study. The concept of the leader's secret room broadens caritative leadership theory and gives insight into the leader's need for a place to retreat for reflection and decision making. This room can also be experienced as a place of loneliness, with a sense of belonging neither to the staff nor to administration. An organization practicing caritative leadership at all levels may be more resilient and effective and may benefit leaders as well as those who are led by focusing on healing and nurturing the leaders themselves as well as other staff members and the institution.^2^,^26^ Studies reflect various motivations to assume leadership positions ranging from a choice due to their desire to care for others^32^ to the path of chance,^30^,^31^ which also has an impact on leaders' ability to facilitate the best possible care in their units.

**Figure 2.**
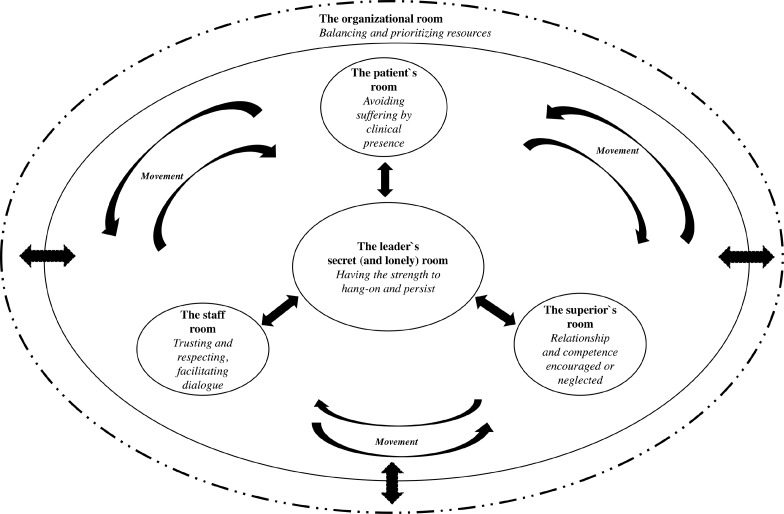
Model illustrates caring in nursing leadership is a conscious movement between “rooms” in the leader's “house” of leadership.

The arguments for using meta-ethnography are as follows: the ability to address the information, exposition, and knowledge fragmentation; the opportunity to identify gaps; the provision of another methodology to advance theories; the possibility of adding depth dimensions to a qualitative study; and the efficiency of a cost-effective approach to research.^34^ Noblit and Hare^35^ state that the validity of meta-ethnographies is related to clarification and resolution rather than observation of inconsistencies and tensions between synthesized materials. However, metasyntheses are also criticized because of a stripping of context; the researchers' use of already interpreted data instead of primary data; and restriction to already available data. There is a tension between combining and synthesizing studies, and maintaining the uniqueness of each study. By remaining close to the text, debating interpretation, and keeping every study in sight, the authors have tried to preserve the significance during the synthesis process.^40^,^41^ See Figure 2.

## IMPLICATIONS

This thorough literature search did not find any metasynthesis identifying nurse leaders' perspective on caring in nursing leadership. From this study, a model was developed abductively describing leaders' perspectives of caring in their leadership. These emanate from patient needs and provide an alternative path for leadership, with the possibility of informing both decision makers and those who plan educational programs to help leaders cope and persist with complex, multifaceted roles. It is a tentative model that needs to be tested. The nursing profession needs more knowledge and evidence for improved nursing leadership.

## CONCLUSION

This model can enhance caring leadership by highlighting significant factors that contribute to development of the best possible care for patients, while balancing the needs of the staff, organizations, and leaders themselves. Nurse leaders possess a unique perspective for developing and enhancing nursing care. Leaders need a solid competence in nursing leadership so that they can balance multiple demands in organizations for the good of all stakeholders in health care.
